# Editorial: Bio-based solutions for sustainable development of agriculture, volume II

**DOI:** 10.3389/fpls.2024.1510226

**Published:** 2024-11-14

**Authors:** Eduardo V. Soares, Spyridon A. Petropoulos, Helena M. V. M. Soares

**Affiliations:** ^1^ Bioengineering Laboratory, ISEP, Polytechnic of Porto, Porto, Portugal; ^2^ CEB−Centre of Biological Engineering, University of Minho, Braga, Portugal; ^3^ LABBELS – Associate Laboratory, Braga/Guimarães, Portugal; ^4^ Department of Agriculture Crop Production and Rural Environment, University of Thessaly, Volos, Greece; ^5^ REQUIMTE/LAQV, Departamento de Engenharia Química, Faculdade de Engenharia, Universidade do Porto, Porto, Portugal

**Keywords:** biological fertilizers, biostimulants, enhance of nutrient uptake, environmental stress, inhibition of phytopathogens (biocontrol), microbial inoculants, microbial siderophores, mycorrhizal fungi

The modern cropping industry has to address food security challenges related to the increasing world population, the pressure of climate change on crop production, and the adverse effects of anthropogenic activities on agro-ecosystems (Boix-Fayos and de Vente, 2023; Grigorieva et al., 2023). Therefore, various strategies have been suggested (e.g. Green Deal, Soil Deal, Farm to Fork, and European Biodiversity) focusing on a gradual and mild transition toward sustainable cropping systems and agronomic solutions. Biostimulants and bio-based solutions in general have been considered viable solutions to be integrated into the current agronomic schemes to overcome the challenges that the cropping sector is facing or is expected to face in the near future.

In this context, this Research Topic invited contributions related to the development of alternative bio-based solutions (e.g. biofertilizers, biostimulants, or biocontrol agents) and their effects on plant growth, physiological processes, quality features of the final product, and tolerance to biotic and abiotic stressors, as well as contributions that provide novel information regarding the mechanisms that underlie the suggested effects.

In the work of Tang et al., a literature review regarding the application of antimicrobial peptides as a means of sustainable crop protection and disease management was performed. The authors summarized the recent reports on the positive aspects of using antimicrobial peptides, including their steep dose-response relations, ability to rapidly kill pathogens, broad synergistic effects with other antimicrobial agents and the immune system of plants, and the slow selection of pathogen resistance. Based on these effects, possible applications of antimicrobial peptides and their integration into sustainable plant protection strategies were also discussed. In addition, Santos et al. reviewed the progress of various strategies to promote sustainable management of Kiwifruit Bacterial Canker (KBC), caused by the bacterium *Pseudomonas syringae* pv. *actinidiae* (Psa). In this paper, the authors focused on two aspects: (i) precision agriculture, where early non-invasive methods to detect the disease and forecast models to predict the risk of Psa infection were addressed, and (ii) biotechnological tools, with emphasis on plant breeding, aiming at Psa tolerance and the use of several novel substances of natural origin to combat Psa. To assess the beneficial effect of fungi in improving plant defense against pathogenic agents, Meesters et al. inoculated tomato (cv. Moneymaker) seeds with the fungi *Beauveria bassiana*, *Metarhizium brunneum*, and *Trichoderma harzianum*. The study revealed that the three fungi had a marginal effect on the volatile organic compounds profile of tomato plants. Nonetheless, the authors observed that the insect *Nesidiocoris tenuis* was deterred on plants inoculated with *M. brunneum*, compared to those non-inoculated (control), which opens up good prospects for using this fungus in new biocontrol strategies against *N. tenuis*. In another work, the antifungal activity of 6-deoxy-6-aminochitosan in the protection of tomato plants against *Botrytis cinerea* was studied for the first time by Moola et al. The authors tested various concentrations of aminochitosan in two scenarios: (i) on *B. cinerea* growth and sporulation (*in vitro*) and (ii) as a foliar pre-treatment in tomato leaves (*in vivo*). *Botrytis cinerea* infection was prevented when aminochitosan was applied as a foliar spray to 5- week-old tomato leaves and the *in vivo* biological activity was improved when aminochitosan was directly applied and when used as a systemic treatment for up to 30 days after inoculation. Zhang et al. investigated the inhibitory effect of aqueous extracts of *Trifolium repens* L. (white clover) and *Lolium perenne* L. (ryegrass) manures on the germination and growth of *Eleusine indica* L. (goosegrass). Highly concentrated (≥ 30%) extracts or decomposed liquids of both manures inhibited the germination of goosegrass, making the ryegrass treatments more effective than the white clover treatments. However, further studies are needed to understand the effects of the decomposition residues on modifications of nutrients, microorganisms and the composition of the soil.

The literature review performed by Khan focused on the interaction of beneficial phytomicrobiome species under stress conditions and how they can enhance plant growth and crop productivity by improving nutrient uptake, the biosynthesis of secondary metabolites and plant resistance to pathogens and abiotic stressors. Moreover, special attention was given to the interaction of the bacterial and fungal biome with archaea, viruses, oomycetes, protozoa, algae, and nematodes, as well as to understanding the stress signaling mechanisms and the responses of phytomicrobiome species under the climate change conditions on organism and ecosystem levels.

In the review report of Rétif et al., the use of seed fungal endophytes as biostimulant and biocontrol agents was investigated focusing on their capacity to improve seed quality and benefit seed biology, while the mechanisms involved in the physiological processes of the improvement of seed performance were also assessed. On the other hand, Swiatczak et al. investigated the effect of six strains of plant growth–promoting rhizobacteria (PGPR) in both sterile and non-sterile conditions on the growth of canola. Additionally, the genomic profile of the two PGPR strains (*Pseudomonas sivasensis* 2RO45 and *Peribacillus frigoritolerans* 2RO30) that demonstrated the highest growth-promoting effect was compared. The authors verified that *P. sivasensis* 2RO45 was enriched with additional genes responsible for ACC deaminase and siderophore production, evidenced antifungal effect against the phytopathogens tested, and presented various biosynthetic gene clusters compared to *P. frigoritolerans* 2RO30. In the work of Carrascosa et al., the effect of various types and doses of fertilizers (inorganic and compost tea) on the growth of Purslane (*Portulaca oleracea* L.) as well as soil quality parameters and their microbial community structure were studied. The first insights of this study pointed out that the fungal and bacterial diversity and loss of bacterial function decreased for the majority of the fertilizer treatments, which needs to be confirmed by long-term experiments and samples collected at different seasons.

Leporino et al. studied the foliar application of two protein hydrolysates (PH) obtained from Malvaceae (PH1) or Fabaceae (PH2) on the tolerance of tomato plants to repeated drought stress. The application of PH1 was more efficient in inducing plant resistance to water stress, while PH2 was only adequate for the first water stress event. PH1 increased the production of the compounds associated with the primary and secondary metabolism of the plant. However, compared to the control, the authors observed a down-regulation of hormones and signaling molecules or molecules involved in the fight against oxidative stress. On the other hand, Hamoud et al. studied the application of calcium lignosulfonate (CLS) to mitigate the stress associated with irrigation of soil with saline water. Soils amended with CLS presented improved chemical characteristics, which had a positive impact on nutrient absorption by maize (*Zea mays*) plants. The application of CLS led to less cellular damage (due to the reduction of oxidative stress), greater plant growth (increased root and shoot length), maize yield, and grains with higher starch, fat, and protein content.

Finally, Zhong et al. investigated the mechanisms associated with *Lilium brownii* F. E. Brown ex Miellez var. viridulum Baker (Longya lily) autotoxicity. The analysis of plant tissues revealed the presence of 2,4-di-tert-butylphenol (2,4-DTBP) as the autotoxic substance. Genes linked with phytohormones, reactive oxygen species, and MAPK signaling cascades were significantly changed in response to 2,4-DTBP. The autotoxic substance also affected chloroplasts and ribosome integrity. The information obtained on the mechanisms underlying the autotoxicity of *L. brownii* may be useful in managing the obstacles associated with its successive cultivation.

In summary, this second volume of the Research Topic brings together 12 works that present new advances in biocontrol and the production of biofertilizers and biostimulants to improve the quality and nutritional value of crops, disease resistance, and resilience to environmental stressors. The new and relevant information presented here, together with those presented in the first volume of this Research Topic (Soares et al., 2022) can be useful in establishing best practices that can lead to the development of modern and more sustainable agriculture.
